# Efficacy of *Syzygium aromaticum* essential oil on the growth and enzymatic activity of pathogenic *Candida albicans* strains

**DOI:** 10.18502/cmm.8.1.9209

**Published:** 2022-03

**Authors:** Ashkan Hekmatpanah, Aghil Sharifzadeh, Hojjatollah Shokri, Sepideh abbaszadeh, Donya Nikaein

**Affiliations:** 1 Department of Microbiology and Immunology, Faculty of Veterinary Medicine, University of Tehran, Tehran, Iran; 2 Department of Pathobiology, Faculty of Veterinary Medicine, Amol University of Special Modern Technologies, Amol, Iran; 3 Health Research Center, Lifestyle Institute, Baqiyatallah University of Medical Sciences, Tehran, Iran; 4 Department of Nutrition and Food Hygiene, Faculty of Health, Baqiyatallah University of Medical Sciences, Tehran, Iran

**Keywords:** *Candida albicans*, *Dianthus caryophyllus*, Enzymatic activity, Fluconazole, Growth inhibition

## Abstract

**Background and Purpose:**

*Candida albicans* (*C. albicans*) is the most common human pathogen owing to the most virulence factors. It seems that extracellular hydrolytic enzymes play a key role in *C. albicans* pathogenicity. The present study aimed to assess the susceptibility and enzymatic activity of pathogenic *C. albicans* isolates exposed to the *Syzygium aromaticum* (*S. aromaticum*) essential oil.

**Materials and Methods:**

*S. aromaticum* oil was characterized using gas chromatography-mass spectrometry (GC–MS). The broth microdilution technique (CLSI, M27-A3) was used to determine the minimum inhibitory concentration (MIC) of test compounds. Furthermore, before and after treatment with *S. aromaticum* essential oil, the yeasts were analyzed regarding the proteinase (Prz), hemolysin (Hz), and phospholipase (Phz) production/activity.

**Results:**

β-caryophyllene (12.76%) was found to be the major constituent in the essential oil after eugenol (84.64%). Only one isolate of *C. albicans* showed the antifungal resistance to fluconazole. All isolates were susceptible to *S. aromaticum* essential oil with MIC of 625-1250 μg/ml. *S. aromaticum* oil represented the best antifungal effect against *C. albicans* at MIC 1000 μg/ml. The mean±SD enzyme activity of *C. albicans* not exposed to *S. aromaticum* essential oil was obtained at 0.55±0.03, 0.73±0.04, and 0.61±0.05 for proteinase, hemolysin, and phospholipase, respectively. The activities of these enzymes were reduced significantly (*P*<0.05) to 0.33±0.06, 0.40±0.04, and 0.16±0.03 for phospholipase, proteinase, and hemolysin, respectively, after the yeasts were subjected to *S. aromaticum* essential oil.

**Conclusion:**

The present study aimed to determine the ability of *S. aromaticum* essential oil to prevent the growth of *C. albicans* and decrease their enzymatic activity. As a natural antifungal agent, *S. aromaticum* can be utilized in pharmaceutical systems.

## Introduction

Recently, the increase in the immunocompromised patient population has increased the occurrence of both systemic and local infections caused by *Candida* spp. In these patients, the yeasts passing through the mucosa can attack various tissues and cause considerable damage and even mortality [ 1 ]. Therefore, various hydrolytic enzymes are secreted by *Candida albicans* (*C. albicans*) to ensure its penetration into the host cells. Various studies have concentrated on assessing fungal lipases and their contribution to fungal pathogenicity [ 2 - 5 ]. Moreover, *C. albicans* adherence is facilitated by the secretion of aspartyl proteinases (SAPs) to numerous host tissues. Assessments of vaginal and oral clinical isolates of *C. albicans* revealed a positive association between the virulence of *C. albicans* and the level of Sap production *in vitro* [ 6 , 7 ]. Moreover, Candida can supply the iron needed for its growth through the production of hemolysin, so the production of hemolysin is essential in the pathogenesis of *Candida* and its ability to survive [ 8 ].

Regardless of the discovery of potent antifungals, most of these infections still present a serious medical problem owing to increasing fungal resistance [ 9 ]. This highlights the need for developing novel therapeutic approaches through searching for agents with new mechanisms of action independent of or together with conventional medicines. Different bioactive molecules have been reported to be obtained from various natural resources as potent antifungal compounds. In our search for natural antifungal compounds from plants, we conducted a phytochemical screening on *Syzygium aromaticum* (L.) flowers (*S. aromaticum *) (carnation), which is one of the flower crops extensively cultivated in Iran [ 10 ]. According to some microbial studies, the *S. aromaticum* essential oil has considerable inhibitory effects against bacterial, fungal, and viral strains [ 11 , 12 ]. Therefore, the present study aimed to determine the chemical composition and inhibitory effects of *S. aromaticum* essential oil on the growth of pathogenic *C. albicans* isolates and the secretion of their hydrolytic enzymes including phospholipase proteinase and hemolysin that are in charge of invasive features.

## Materials and Methods

### Fungal strains and growth circumstances

The fungal isolates were obtained from the archival collection of the Laboratory of Mycology Research Center, Faculty of Veterinary, University of Tehran, Iran, and included one isolate from the American Type Culture Collection (ATCC 10231) and 15 clinical *C. albicans* isolates. The study protocol was approved by the University Ethics Committee (Code of Ethics: 300511/6/4). All *C. albicans* isolates were identified in previous studies using CHROM agar Candida (CHROM agar *Candida* Company, Paris, France), RapID^TM^ yeast identification system (Remel, USA), and molecular analysis [ 13 ]. 

### Extraction and chemical analysis of S. aromaticum essential oil

*S. aromaticum* flower buds prepared from Pars Iman Daru Company (Tehran, Iran) were exposed to water distillation for 3 h utilizing a Clevenger-type apparatus, according to the procedure reported by Divband et al. (2017) (Table 1) [ 14 ]. The oil was then stored at 4oC in the dark before chemical analysis and mycological tests.

**Table 1:** Ethnobotanical data and active constituents of the studied plant.

Scientific name	English name	Family	Used part	Common Medicinal uses	Active Constituents
*Dianthus caryophyllus*	Carnation, clove pink	CaryophyllaceaeFlower	Skin toner, antifungal, relief of acute dermatitis, tooth pain, vomiting and gastritis, digestive function stimulant, antispasmodic	Flavonoids, anthocyanins,	Dianthramides, Antiretroviral proteins and phenols

Gas chromatography-mass spectrometry (GC–MS) and gas chromatography (GC) were utilized for essential oil analysis. Subsequently, GC was conducted via two fused silica capillary columns with various stationary phases including SPB-1 (ﬁlm thickness of 0.20 μm; polydimethylsiloxane 30 m × 0.20 mm i.d.), and SupelcoWax 10 (ﬁlm thickness of 0.20 μm; polyethyleneglycol 30 m×0.20 mm i.d.); injector temperature: 250°C; detector carrier gas: helium, adjusted to a linear velocity of 30 m s^-1^; oven temperature program: 70–220°C (3°C min^-1^), 220oC (15 min); splitting ratio 1: 50; temperatures: 250°C. To perform GC–MS, an HP1 fused silica column was used (ﬁlm thickness of 0.25 μm; polydimethylsiloxane 30 m × 0.25 mm i.d.), interfaced with a mass selective detector. GC parameters included interface temperature: 250°C; MS quadrupole temperature: 150°C; MS source temperature: 230°C; ionization current: 60 μA; ionization energy: 70 eV; the scan range: 35–350 μm; and scans: s^-1^:4.51. Ingredients were recognized by their retention indices that were calculated by linear interpolation relative to retention times of some n-alkanes, as well as their mass spectra, and were compared with those in published studies [ 2 ]. Relative quantities of individual components were determined based on GC peak areas without FID response factor correction.

### Antifungal activity assay

The susceptibility of *C. albicans* isolates to fluconazole and *S. aromaticum* was determined using Roswell Park Memorial Institute 1640 medium (Sigma, St Louis, MO, USA) buffered to pH 7.0 with 0.165 M 3-(N-morpholino) propanesulfonic acid (MOPS) buffer. A minimum inhibitory concentration (MIC) assay was conducted utilizing the standardized broth microdilution practice (M27-A3-S4) of the Clinical and Laboratory Standards Institute [ 15 ]. Briefly, dilution of fluconazole was performed in the RPMI-1640 in the above-described medium in 96-well microtitre plates to ultimate concentrations (128 μg/ml- 0.125 μg/ml). Moreover, different dilutions of *S. aromaticum* essential oil were prepared (39, 78.1, 156.2, 312.5, 500, 625, 1000, 1250, 2000, 2500, and 5000 μg/ml) in 96-well microtiter plates through MOPS-buffered RPMI-1640 media (Sigma, St. Louis, MO, USA). Isolates were added to achieve an ultimate concentration of 0.5-2.5×10^3^ cfu/ml. Positive and negative controls consisted of wells without antifungals and microorganisms, respectively. The incubation of the microtitre plates was performed for 48 h at 30oC. The MIC was interpreted as the lowest concentration of antifungal agent that totally inhibited the growth of the tested Candida strains compared with the control, after the incubation time. Each experiment was performed thrice. Moreover, the media from wells with yeasts presenting no visible growth was further cultured on Sabouraud dextrose agar (SDA) (Merck, Darmstadt, Germany) to determine the minimum fungicidal concentration (MFC). Following the determination of the MFC value as the lowest concentration, only four colonies were yielded, corresponding to 98% mortality of the yeasts in the primary inocula.

For each isolate, fluconazole breakpoints were allocated as susceptible dose-dependent (SDD), MIC=4 μg/ml; susceptible (S), MIC≤2 μg/ml; resistant (R), MIC ≥ 8 μg/ml, utilizing the M27-A3 protocol for all isolates [ 15 ]. *C. parapsilosis* ATCC 22019 and *C. krusei* ATCC 6258 were used as reference strains. 

### Pre-treating C. albicans cells with S. aromaticum essential oil

The *S. aromaticum* essential oil at MIC was applied on fresh suspensions of *C. albicans* isolates for 1 h, at 35°C (in RPMI-1640 liquid medium). They were then rinsed three times with phosphate-buffered saline (PBS) to avoid carryover effects [ 16 ]. The prepared *Candida* suspensions were utilized twice in the assays on three separate occasions described in the following.

### Measurement of hemolysin, phospholipase, and proteinase activities of *C. albicans* treated with oil

An egg yolk agar assay was used to examine phospholipase activity (Phz). The test medium comprised SDA supplemented with 1M CaCl_2_, 1M NaCl, and egg yolk (10%) [ 17 ]. Bovine serum albumin (BSA) assay was used to evaluate proteinase activity (Prz). The medium included BSA (0.2%), yeast extract (0.01%), glucose (1.17%), and agar (2%) at pH 5.0 [ 18 ]. The assay of Hemolytic activity (Hz) was performed on SDA plates supplemented with 7% horse blood [ 19 ]. Inoculation of an aliquot (5 μl) of the yeast suspension preincubated with essential oil was performed on the plates comprising the above substrates for 3 days (phospholipase, proteinase) or 2 days (hemolysin) at 37°C. Staining BSA plates with amido black - 0.25% (w/v) was performed in glacial acetic acid 49.75% (v/v) before measuring the proteinase activity. The plates were then washed immediately with distilled water. Phz, Hz, and Prz were presented as the proportion of *C. albicans* colony diameter in comparison with the mean precipitation/hemolysis zone’s diameter±standard deviation (SD). Based on the applied contractual scale, Phz, Hz, and Prz values of 1 revealed negative reaction, while the values of 0.9-0.99, 0.8-0.89, 0.7-0.79, 0.6-0.69, and <0.59 showed very weak, weak, moderate, strong, and very strong secretory activity, respectively [ 2 ].

### Statistical analysis

Utilizing the t-Student test (Sigma Stat, version 3.5), all results were statistically analyzed, and the level of statistical significance was set at 0.05.

## Results

Based on the GC-MS analysis, it was indicated that the scent compounds of *S. aromaticum* comprised of 14 volatile compounds (Table 2), of which one benzenoid, including eugenol (84.64%), was the major component in essential oil after one terpenoid including β-caryophyllene (12.76%).

**Table 2:** Chemical composition of *Dianthus caryophyllus* essential oil

No.	Retention time	Compound	Percentage	Purity	Reference number
1	10.24	1,8- cineole	0.02	95	000470-82-6
2	13.363	Linalool - L	0.02	87	000078-70-6
3	17.589	Methyl salicylate	0.08	96	000119-36-8
4	26.627	Eugenol	84.64	98	000097-53-0
5	26.961	β-elemene	0.12	95	000515-13-9
6	27.449	Isocaryophyllene	0.19	99	013877-93-5
7	27.587	Methyl eugenol	0.04	97	000093-15-2
8	28.116	β-caryophyllene	12.76	99	000087-44-5
9	29	Humulen	0.02	99	000000-00-0
10	29.138	α- humulene	0.84	98	006753-98-6
11	29.801	δ- cadinene	0.03	93	000483-76-1
12	31.47	Calamenene	0.10	96	000483-77-2
13	33.365	Caryophyllene oxide	0.54	91	001139-30-6
14	37.95	Conifero	l0.21	72	000458-35-5
Total	99.59				

Table 3 presents the anti-*Candida* activity of the *S. aromaticum* essential oil based on MIC and MFC. It was found that S. aromaticum oil was active against the examined yeasts at concentrations <1250 μg/ml. The MICs of *C. albicans* were within the ranges of 0.25-32 μg/ml and 625-1250 μg/ml for fluconazole (mean±SD: 3.04±0.21 μg/ml) and *S. aromaticum*, respectively (mean±SD value: 953.13±218.30 μg/ml). Moreover, MFCs of *C. albicans* were within the range of 1-64 μg/ml (mean±SD value: 9.37±1.46 μg/ml) for fluconazole and 1000-2500 μg/ml (mean±SD value: 1921.90±582.50 μg/ml) for *S. aromaticum*.

**Table 3:** Minimal fungicidal concentration (MFC) and minimal inhibitory concentration (MIC) values of fluconazole and *Syzygium aromaticum* essential oil in *Candida albicans*

Isolate	Source	Fluconazole		*S. aromaticum* essential oil	
		MIC (μg/ml)	MFC (μg/ml)	MIC (μg/ml)	MFC (μg/ml)
1	Animal	0.25	1	1000	2000
2	Animal	0.25	2	1000	2500
3	Animal	0.5	2	625	1250
4	Animal	0.5	2	1000	2000
5	Animal	1	4	625	1250
6	Animal	2	16	1250	2500
7	Animal	0.25	1	1250	2500
8	Animal	0.25	1	1000	2000
9	Animal	4	16	625	1000
10	Animal	2	16	1000	2500
11	Human	0.5	4	1000	2500
12	Human	0.5	2	1250	1250
13	Human	0.25	1	1000	2000
14	Human	4	16	625	1000
15	Human	32	64	1000	2500
*C. krusei*	ATCC 6258	0.5	2	1000	2000

An auspicious method was adopted in some studies on *C. albicans* virulence factors for creating novel treatment targets. The examination of agar plates comprising substrates for particular enzymes, such as horse blood, egg yolk, or BSA, showed that *C. albicans* isolates examined in our work had different quantities of produced enzymes. Moreover, *C. krusei* ATCC 6258 was positive for the strong production of proteinase, phospholipase, and hemolysin (Prz=0.64±0.04, Phz=0.61± 0.02, and Hz=0.78±0.01). 

**Figure 1:**
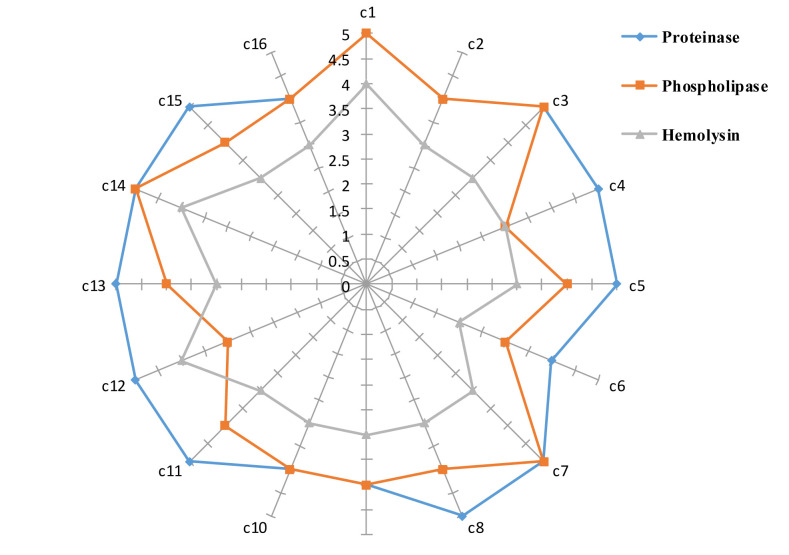
Proteinase, phospholipase, and hemolytic activities of each *C. albicans* isolate before treatment with Syzygium aromaticum essential oil (*C. albicans* isolates 1-16: C1-C16)

It was found that 68.7% and 31.2% of *C. albicans* isolates were very strong and had strong proteinase activities, before treating *C. albicans* isolates with *S. aromaticum* essential oil. Moreover, 25% and 56.2% of *C. albicans* isolates were very strong and had strong phospholipase activities, respectively. However, strong hemolytic activity was found in 18.7% of *C. albicans* isolates. The mean±SD phospholipase, proteinase, and hemolytic activities of all the isolates were obtained at 0.61±0.05, 0.55±0.03, and 0.73±0.04, respectively (Figure 1).

**Figure 2:**
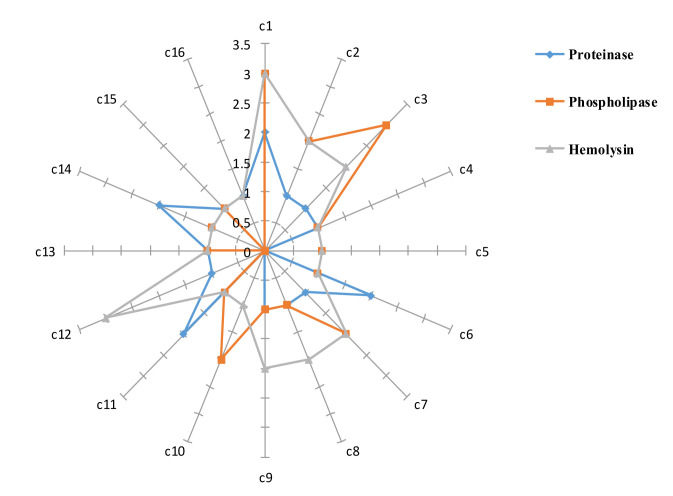
Proteinase, phospholipase, and hemolytic activities of each *C. albicans* isolate after treatment with Syzygium aromaticum essential oil (*C. albicans* isolates 1-16: C1-C16)

A statistically significant reduction was found in the release of tested enzymes through analysis of the enzymatic activity of *S. aromaticum* oil-treated *C. albicans* isolates. In total, 62.5%, 56.2%, and 56.2% of *C. albicans* isolates showed significantly weak proteinase, hemolytic, and phospholipase activities, respectively (*P*<0.05). Moreover, the mean±SD phospholipase, proteinase, and hemolytic activities of the isolates were reported to be 0.33±0.06, 0.40±0.04, and 0.16±0.03, respectively (Figure 2).

## Discussion

In Iran, the same as many other countries, the plants with health benefits were picked up and utilized for treating different diseases. The chemical compounds, as well as anti-enzymatic and antifungal properties of *S. aromaticum* essential oil against pathogenic *C. albicans* was determined in the present study. Moreover, the major components of *S. aromaticum* included eugenol and β-caryophyllene. According to a former study in Turkey [ 20 ], the chemical composition of *S. aromaticum* oil approximately contained eugenol (87%), β-caryophyllene (3.56%), and eugenyl acetate (8.01%). In other studies performed by Porta et al. (1998), Lee and Shibamoto (2001), and Tomaino et al. (2005), the results obtained by GC-MS analysis indicated that eugenol (82.60%), eugenol (89.20%), and eugenol acetate (77.40%) were the major components of S. aromaticum oils, respectively. Our results were consistent with the reported values [ 21 - 23 ].

In this study, most *C. albicans* isolates (93.5%) were susceptible to fluconazole. According to the results of the study conducted by Duarte et al. (2007), MIC of 500 μg/ml, within 501-1000 μg/ml, and ≥1001 μg/ml indicated strong, moderate, and weak inhibitory essential oil, respectively. The S. aromaticum essential oil presented a moderate inhibitory effect on 81.25% of pathogenic *C. albicans* [ 24 ]. Our results regarding the antifungal activity of *S. aromaticum* were consistent with those reported by Shahidi Bonjar (2004) and Dababneh (2008) [ 11 , 25 ]. They utilized *S. aromaticum* taken from commonly used medicinal plants against pathogenic *C. albicans* with MIC values of 1250 μg/ml and 800 μg/ml, respectively. Erturk (2006) found that the ethanolic extract obtained from *S. aromaticum* presented weak inhibitory activity against the fungus *Aspergillus niger* (*A. niger*) (MIC value of 25000 μg/ml) and the yeast *C. albicans* (MIC value of 20000 μg/ml), compared to the standard antifungal fluconazole [ 26 ]. In another study, it was revealed that methanolic, ethanolic, and water extracts taken from *S. aromaticum* had antifungal activities compared to hexane extract [ 27 ]. They showed that *A. niger* isolates were inhibited by *S. aromaticum* extracts at concentrations between 62.5-250 μg/ml, using the broth microdilution method. Furthermore, the methanolic extract had strong antifungal activity against *A. niger* using the disc diffusion method. It represented the diameter of the zone of inhibition (24 mm) while water and ethanolic extracts revealed the diameter of the zone of inhibition (21 and 22 mm), respectively. Hexane extracts of *S. aromaticum* showed very low antifungal activity against *A. niger* ATTC16404 (diameter of inhibition zone 14 mm) [ 27 ]. Picman et al. (1995) and Galeotti et al. (2008) also reported fungitoxic properties of *S. aromaticum* against *Fusarium oxysporum* and *Verticillium alba-atrum*, respectively [ 12 , 28 ].

The existence of several components, mainly eugenyl acetate, eugenol, and β-caryophyllene reveals the inhibitory activity of *S. aromaticum* [ 29 ]. Yang et al. (2003) mentioned the inhibitory activity of *S. aromaticum* oil/extracts in the presence of acetyl eugenol, methyl salicylate, α-humulene, isoeugenol, and methyl-eugenol. Such phenolic structure components as eugenol have higher activity against the fungi [ 30 ]. Consistently, the results in this study showed a high percentage (84.64%) of eugenol in *S. aromaticum* oil. This group is known as either fungicide or fungistatic agents, based on the concentration reported by Abbaszadeh et al. (2014) and Morcia et al. (2012) [ 31 , 32 ]. These compounds had strong activity regardless of relatively low dissolvability in water. This result is consistent with the published data since the phospholipid bilayer of the fungal cytoplasmic membrane is sensitized by these compounds, leading to the incremented permeability, impairment of fungal enzyme systems, or unavailability of vital intracellular constituents [ 33 ].

To the best of our knowledge, this is the first study in which *S. aromaticum* oil has impaired the formation of phospholipase, proteinase, and hemolysin of pathogenic *C. albicans*. On the other hand, relatively little research was performed on the effects of essential oils and other natural substances on producing hydrolytic *C. albicans* enzymes. Budzyńska et al. (2014) showed that both *C. albicans* ATCC 90028 and *C. albicans* ATCC 10231 were positive for producing extracellular proteinase (Prz=0.27±0.01; Prz=0.34±0.01, respectively), phospholipase (Phz=0.44±0.02), and hemolysin. However, the former’s activity was evaluated as medium (Hz=0.57±0.03; Hz=0.62±0.04). It was shown that geranium oil, clove oil, citronella oil, and lemon balm, even at a sublethal concentration (½ MIC), exhibited considerable biological activities that decrease the production of these enzymes [ 16 ]. Silva-Rocha et al. (2015) added the crude extract of *Eugenia uniflora* to proteinase and phospholipase media. They found that the activity zone of the enzymes was either completely inhibited or highly reduced [ 34 ]. Rajkowska et al. (2014) in another study demonstrated alterations in cell morphology and C. albicans colony. Moreover, they reported the reduction of enzymatic activity in thyme and tea tree oils in most cases [ 35 ]. Pootong et al. (2017) also reported that the mean±SD Prz and Phz for *C. albicans* not exposed to cinnamaldehyde was obtained at 2.17±2.17 and 1.98±0.46, respectively. The activities of these enzymes were reduced significantly (*P*<0.01) when the yeast was exposed to cinnamaldehyde at 62.5 μg/ml and 31.25 μg/ml, reaching 1.45±0.27 and 1.92±0.17 for the proteinase and 1.40±0.38 and 1.65±0.49 for the phospholipase, respectively [ 36 ]. Yordanov et al. (2008) found that the cultivation of *C. albicans* while existing four natural substances at various concentrations led to a dose-based reduction of phospholipase activity. Ibogaine (75.0%±6.2% inhibition) had the best inhibition effect at 250 mg/ml concentration after berberine (up to 60.0%±4.5% inhibition) and kaempferol (up to 40.0%±2.0% inhibition) [ 37 ].

Well-known and important virulence factors secreted from *C. albicans* cells include hydrolytic enzymes, such as phospholipases and proteinases. These enzymes have a key role in adhesion to host cells, nutrition, and tissue destruction leading to the pathogen spread. Among these virulence factors, Saps are the most important. *Candida* blastospores secrete Saps1-3, while Saps 4-6 are mainly released by filamentous forms, and Sap 9 and 10 are strongly associated with the cell wall of both morphotypes. The phospholipase action complements the protease activity as enzymes that are in charge of hydrolyzing one or more ester bonds in the cell membrane glycerophospholipids. Another vital virulence feature of *C. albicans* is the effective acquisition of iron presented by the action of some proteins with mannan/mannoproteins and hemolytic activity-Rbt5, released from the cell wall [ 38 , 39 ]. 

A comparison of the susceptibility to fluconazole and enzyme production revealed that the susceptible *C. albicans* strains to antifungal agent utilized in this study represented a robust positive enzymatic activity to phospholipase, proteinase, and hemolysin. This finding is vital since the growth of pathogenic *C. albicans* isolates is inhibited by herbal essential oils, such as *S. aromaticum*, which possibly reduce the enzymatic production. The contribution of such enzymes in disease progression and initiation is still largely unrecognized; however, it seems that the disease severity can be affected by their expression level in *Candida* infections [ 12 ]. In this regard, a correlation was observed between the existence of oral [ 8 ], vulvovaginal [ 7 ], and pulmonary [ 40 ] candidiasis and the secretion of extracellular enzymes.

## Conclusion

Based on the obtained results in this study, most of the pathogenic *C. albicans* isolates were susceptible to *S. aromaticum* essential oil and fluconazole and showed high phospholipase, proteinase, and hemolytic activities. These enzymes’activities were reduced significantly by exposing the yeasts to *S. aromaticum* oil. Eventually, it can be assumed that *S. aromaticum* can significantly affect *C. albicans* pathogenicity through these enzymatic changes and loss of growth capability.

## Acknowledgments

This research work was supported/funded by the University of Tehran, Tehran, Iran.

## Authors’ contribution

Study conception and design were conducted by A.S., D.N., and H.S. Data were collected by A.H. and D.N. Analysis and interpretation of results was performed by A.H., A.S., and S.A. Manuscript draft was prepared by H.S., A.S., and S.A. All authors reviewed the results and approved the final version of the manuscript. 

## Conflicts of interest

No conflict of interest was declared by the authors.

## Financial disclosure

The authors have no financial interests regarding the material in the manuscript.
